# Deviants Are Detected Faster at the End of Verse-Like Sound Sequences

**DOI:** 10.3389/fpsyg.2021.614872

**Published:** 2021-08-31

**Authors:** Varun D. C. Arrazola

**Affiliations:** ^1^Leiden University Centre for Linguistics, Leiden, Netherlands; ^2^The Meertens Institute, Amsterdam, Netherlands

**Keywords:** auditory oddball, entrainment, attention, poetry, song, verse

## Abstract

Songs and poems from different traditions show a striking formal similarity: lines are flexible at the beginning and get more regular toward the end. This suggests that the free-beginning/strict-end pattern stems from a cognitive bias shared among humans. We propose that this is due to an increased sensitivity to deviants later in the line, resulting from a prediction-driven attention increase disrupted by line breaks. The study tests this hypothesis using an auditory oddball task where drum strokes are presented in sequences of eight, mimicking syllables in song or poem lines. We find that deviant strokes occurring later in the line are detected faster, mirroring the lower occurrence of deviant syllables toward the end of verse lines.

## 1. Introduction

Verse encompasses a variety of forms, such as song, poetry, chant, or nursery rhymes. All of these contain words, but they also include a feature which is absent from everyday speech, viz. in verse, the selection and combination of words is constrained by templates that control the structure of the line in some way. Often, for instance, the length of lines in poems or songs is limited to a fixed number of syllables, accents or beats. In many languages, the relative prominence of the syllables is also constrained, so that e.g., when creating an English sonnet in iambic pentameter, lines are usually opened with an unstressed syllable, followed by a stressed one; this binary alternation is then kept along the line.

Notwithstanding these structural constraints, verse is not completely rigid, as poets and singers often deviate from the templates, introducing unexpected elements which can be exploited for aesthetic purposes by generating interest or surprise in the listener (Huron, 2006). Studies of verse corpora show that, still, it is possible to generalise *where* deviations tend to occur: they are most frequent at the beginning of lines, and their incidence (progressively) decreases (Fabb, 2002, p. 173–177). This phenomenon is referred to by terms such as *final strictness* or *initial looseness*, bringing out the fact that the asymmetry can stem from exceptional events at either edge of the line.

Despite the lack of a systematic cross-linguistic survey, robust final strictness phenomena are reported for languages from unrelated families, such as Sanskrit (Arnold, 1905), Finnish (Kiparsky, 1968), Tashlhiyt Berber (Dell and Elmedlaoui, 2008), and Greek (Allen, 1973; Golston and Riad, 2000). A statistical analysis on these languages including more than 50k lines of verse (deCastro-Arrazola, 2018) shows that entropy is higher at the beginning of lines, and consistently decreases later in the line. Higher entropy values indicate a higher incidence of deviants; i.e., earlier positions are less consistent in the use of a particular kind of syllable (e.g., stressed vs. unstressed) than later ones. Besides these systematic case-studies, a number of other asymmetries in song and poetry lines also follow this pattern, such as rhyme or melodic cadence. Displaying a regular rhyme at the end of lines is a common feature of many traditions, while the opposite (i.e., systematic alliteration between the beginning of different lines) is exceedingly rare in the languages of the world [although reported e.g., for Mongolian, Krueger (1961); Fabb (1999); Kara (2011) for examples from other languages].

Hence, there is some evidence that final strictness is not a property linked to a limited set of related languages which accidentally developed the tendency. Instead, the range of independent observations of final strictness, and the lack of a robust set of languages showing the opposite pattern (i.e., initial strictness) asks for a common explanation. There is the possibility, for instance, that the effect is driven by some aspect of cognition shared across populations. In the present paper we explore one hypothesis within this context, namely, that the decrease in the frequency of deviations is due to an increase of attention along the line, which is disrupted between lines.

Previous studies show that if the occurrence of an event can be predicted, its processing is facilitated (Niemi and Näätänen, 1981; Jongsma et al., 2004). The internal regularities characteristic of verse lines allow for prediction building to take place. Nevertheless, this process may be disrupted by line boundaries, which are a defining feature of verse (Fabb, 2015). Other constituent levels such as the stanza or the hemistich may also show comparable disruptions, but we focus on the line because it is the constituent for which (1) final strictness is most often described (Fabb, 2002), (2) universality has been argued (Hayes, 1983, p. 388).

In the present paper we use sequences of drum strokes as a model of verse lines. Subjects are asked to detect deviations from a pattern under three different experimental conditions. We manipulate the relative timing of the strokes in order to test the extent to which the regularity of the stimuli and the pause between the sequences is driving the variation in reaction times (RT). This manipulation directly relates to performance practices; certain verse traditions use isochronous, beat-based deliveries (typical of metrical singing), while others use speech-like deliveries with no overt isochrony (typical of recited poetry). By determining how different kinds of temporal regularity relate to the detection of deviants, we may aim at finer-grained hypotheses about final strictness in different verse traditions. Overall, our results show that it takes longer to detect deviations closer to the beginning of the line, mirroring the data from verse corpora. The effect (decreasing RTs) is observed in all experimental conditions, including those lacking isochrony.

## 2. Method

### 2.1. Participants

A total of 53 subjects took part in the experiment, and each was randomly assigned to either condition 1 (*n* = 17), condition 2 (*n* = 17), or condition 3 (*n* = 19). The communication of demographic details (gender, age) was optional, and hence not reported here. All participants, however, belonged to a young adult population having completed secondary education: they were all students at either Leiden University or Radboud University (The Netherlands) at the time of recruitment. The assignment of conditions to participants did not depend on the place of recruitment. All participants signed an informed consent before performing the task (in accordance with Leiden University's LUCL ethics procedure).

### 2.2. Procedure

The general procedure of the experiment was the same for all three conditions, i.e., subjects performed an auditory oddball experiment. Each participant listened to a total of 576 drum strokes divided into 72 eight-stroke experimental trials. The strokes could be of two kinds: (1) a deviant stroke (*n* = 48), or a standard stroke (*n* = 528). Both sounds are publicly available studio recordings of a mridangam drum (Anantapadmanabhan et al., 2013), comparable in frequency and intensity, but with differing timbre.^1^

All participants performed the task in a silent room, using headphones (Beyerdynamic DT 880). Due to technical convenience, two machines were used: conditions 1 and 2 were presented on a Dell XPS M1330 laptop running Ubuntu 12.04, and condition 3 was presented on a Lenovo T440s running Ubuntu 15.10. A common issue when using different technical setups is that each machine presents its own lag or bias, which affects the recorded RTs. However, this bias tends to be systematic, and can be neutralised by normalising the values (Bridges et al., 2020). In our case, the responses recorded on the Lenovo setup were systematically faster (see Supplementary Table 1); the different distributions of raw RTs, however, become comparable when transformed to z-values (Supplementary Figure 1).

**Figure 1:**
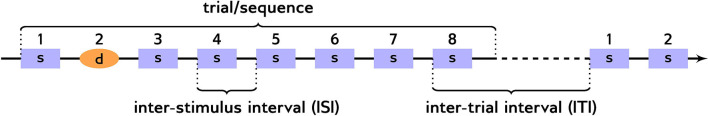
Temporal presentation of the stimuli, with time running from left to right following the arrow. The rectangular 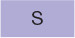 symbol represents a standard drum stroke, and the oval 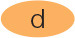 symbol represents a deviant stroke.

Participants were instructed to press the spacebar on the laptop as soon as they detected a deviant stroke. The general temporal configuration of the 576 strokes was similar across conditions: the strokes were played sequentially, with a short silent gap after every stroke (between 250 and 500 ms, depending on the condition, see Table 1), and a longer gap after every eighth stroke (between 1,200 and 1,800 ms, depending on the condition). Figure 1 depicts the temporal presentation of the stimuli, with time running from left to right following the arrow.

**Table 1:** Summary of the parameters which define the three experimental conditions.

**Condition**	**Inter-stimulus interval (ISI) (ms)**	**Inter-trial interval (ITI) (ms)**
1: constant ISI & ITI	300	1,500
2: variable ITI	300	1,200 ~ 1,800
3: variable ISI	250 ~ 500	1,500

As seen in Figure 1, we refer to the group of eight strokes separated by a longer gap as a *sequence* or a *trial*. The duration from the beginning of a stroke to the beginning of the following stroke is called the *inter-stimulus interval* (ISI). The longer gap between sequences is called the *inter-trial interval* (ITI). Each participant listened to a total of 72 sequences, two thirds of which (*n* = 48) contained a deviant, and the remaining third (*n* = 24) served as fillers with no deviant. None of the sequences contained more than one deviant, and deviants were equally probable in each of the eight available positions (i.e., each position contained a deviant in six trials). The key measure taken during the experiment is the reaction time to detect the deviant, i.e., the lapse of time between the onset of the deviant and the subject pressing the key.

All three conditions contain the same number of sequences and deviants, but they differ in their temporal presentation, as summarised in Table 1. The ISI is kept constant (300 ms) in conditions 1 and 2, i.e., strokes within sequences are isochronous. In condition 3, the ISI varies randomly and can take any value between 250 and 500 ms. The difference between conditions 1 and 2 lies in the ITI, which is kept constant (1,500 ms) for condition 1, but varies randomly in condition 2 in the range between 1,200 and 1,800 ms. All random values were generated from a discrete uniform distribution, as implemented in Python's random.randint function. A unique random seed was set for each participant, based on their numeric subject identifier, and new random values were generated continuously across stimuli and trials, preventing thus any regularity.

### 2.3. Statistical Analyses

The main test we performed assesses whether deviants occurring earlier within a sequence were detected more slowly than later deviants. This is based on the observation that deviant syllables are more likely to occur earlier in verse lines, as formulated by the Strict End Hypothesis (deCastro-Arrazola, 2018). Thus, we built a mixed-effects model with reaction time as the dependent variable, deviant position as a fixed effect, and subject as a random effect. The model further includes experimental condition as a predictor, and two additional covariates: deviant distance and deviant probability.

For a given deviant stroke, we define its *deviant distance* as the number of standard strokes between the current deviant and the preceding deviant. While deviants occurring in position 1 are separated from their preceding deviant by a mean of 7.2 (*SD* = 6.87) standard strokes, the deviant distance increases to 15.7 (*SD* = 7.63) in position 8 [*r*_(2089)_ = 0.33, *p* < 0.001]. This difference follows from the fact that each sequence contains a maximum of one deviant, meaning that deviants in position 8 are necessarily preceded by at least seven standard strokes; however, this is not the case for a deviant in position 1, which can be immediately preceded by another deviant. Overall, the deviant distance ranges from 0 to 56 (*M* = 11, *SD* = 7.29). It is important to control for this potential confound, since it correlates with our main predictor (deviant position); to be sure, the number of preceding standards has been shown to play a role in the sensitivity to deviants (Cowan et al., 1993).

The second covariate, deviant probability, captures the fact that positions later in a sequence have a higher probability of containing a deviant. Again, this follows from the fact that each sequence may contain one deviant at most. Two thirds of the experimental trials presented to each participant contain a deviant, which produces a baseline probability of 2/3 (≈ 0.66) for any given trial to contain a deviant. Note, however, that the probability of containing a deviant increases within each sequence, as the number of remaining positions decreases. The deviant probability at position 1 equals 2/3*1/8 (≈ 0.083), at position 2 it equals 2/3*1/7, and so on, until the probability peaks on the 8th position, at 2/3*1/1.

Equation (1) summarises the saturated model, which includes the interaction between experimental condition and the three other fixed effects: deviant position, distance, and probability. The model has been implemented in R (R Core Team, 2021) using the statistical packages lme4 (Bates et al., 2015) and lmerTest (Kuznetsova et al., 2016). Starting with the full model in Equation (1), we conducted a backward model selection based on the AIC statistic (Venables and Ripley, 2013). This procedure drops the terms in the full model in a stepwise fashion, in order to select the most parsimonious model for the dataset.

(1) $$\begin{array}{l} logRT\text{\_}z\sim deviant.position+deviant.distance\\ \;+deviant.probability+condition\\ \;+deviant.position:condition+deviant.distance:condition\\ \;+deviant.probability:condition+\left(1|subject\right) \end{array}$$

Note that, instead of taking raw RTs (in ms) as the dependent variable, we log-transformed and z-normalised the RTs by setup to make them comparable.^2^ Details on this transformation and all the reproducible steps of the analysis can be found in the online Supplementary Material.

## 3. Results

Before analysing RTs, we inspected response accuracy by participant. First, we excluded from the subsequent analyses all incorrect responses, defined as: missed deviants (i.e., participant fails to react to deviant), or false alarms (i.e., participant reacts in filler sequences, or before the onset of the deviant). Whenever a participant reacted more than once to the same deviant, only the first RT was kept. The mean accuracy was comparable across conditions: condition 1 (*M* = 97.6, *SD* = 1.66), condition 2 (*M* = 96.7, *SD* = 2.97), condition 3 (*M* = 97.9, *SD* = 2.33).^3^ Based on this, we excluded all observations from participants whose accuracy was below 90% (seven participants were thus excluded). Finally, we excluded extreme values (such as physically impossible RTs of a few milliseconds) that exceeded two standard deviations from the mean of both the respective participant and the deviant position in question. Supplementary Table 1 summarises the observations retained after filtering: a total of 2,091 observations produced by 46 subjects.

Overall, the recorded RTs were shortest for condition 3, as shown in Supplementary Table 1. However, note that, as explained in section 2.2, differences in experimental setup lead to systematic differences in timing. Hence, all further analyses rely on log-transformed, z-normalised RTs, where all conditions show similar overall distributions (Supplementary Figure 1).

**Figure 2:**
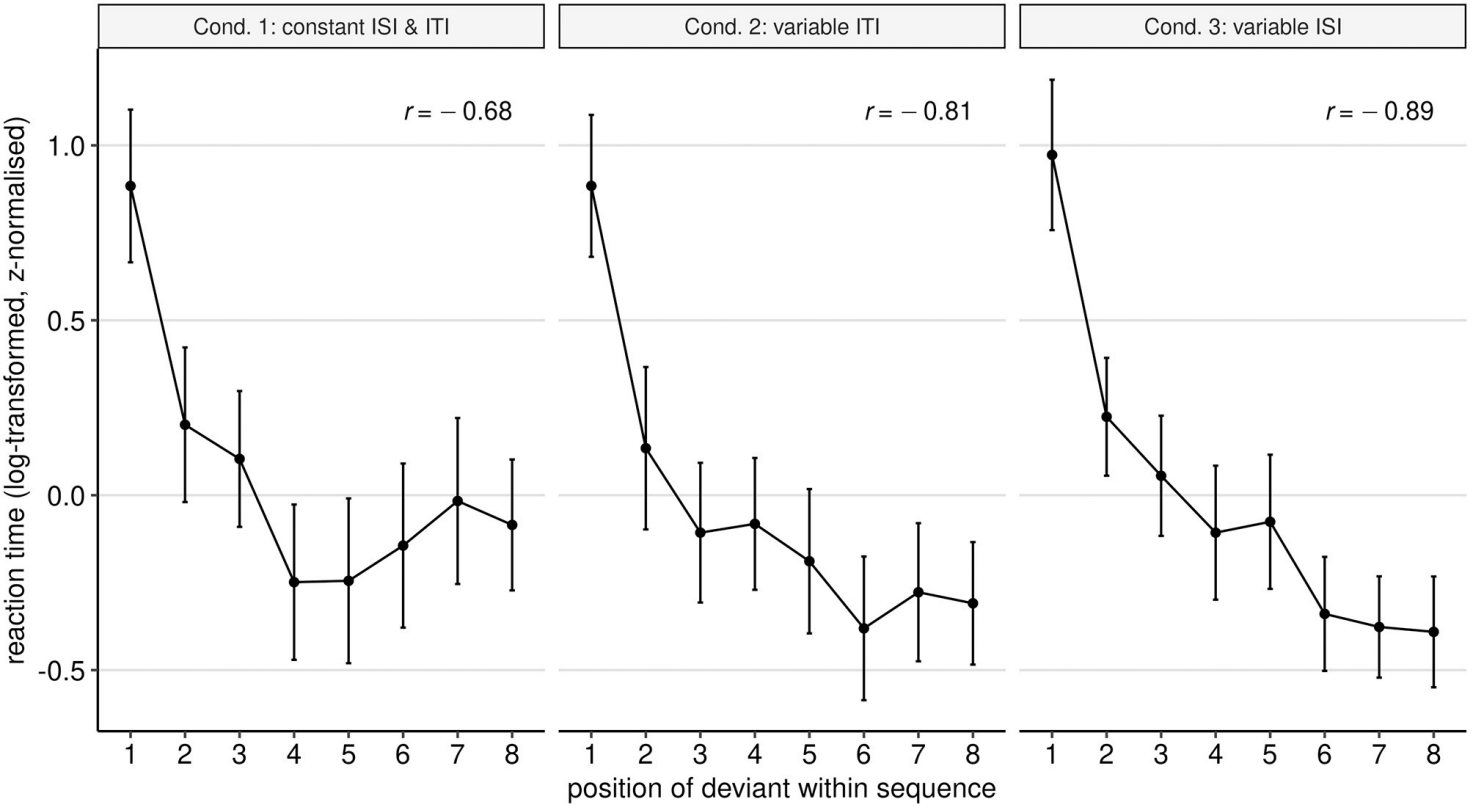
Mean reaction time (RT) to detect the deviant in each of the eight possible positions within the sequence. Raw RT values have been log-transformed and then z-normalised to ensure comparability across experimental setups. The overall correlation between deviant position and RT is indicated by the Pearson's correlation coefficient, which has been computed for each condition separately. Error bars indicate the 95% confidence interval.

Visual inspection of the RTs plotted against the deviant position (Figure 2) reveals a strong negative correlation: deviants occurring later in the line require less time to be detected.

After a backward model selection, the mixed-effects model in Equation (2) was selected. The results of the model are summarised in Table 2 (note that condition 1 is taken as a baseline with which the other two conditions are compared).

(2) $$\begin{array}{l} logRT\text{\_}z\sim deviant.position+deviant.distance\\ \;+deviant.probability+condition\\ \;+deviant.probability:condition+\left(1|subject\right) \end{array}$$

As hypothesised, later deviants were detected faster than deviants in earlier positions (*t* = −15.74, *p* ≤ 0.001). Although less pronounced, a longer distance between deviants also predicts a decrease in RT (*t* = −3.88, *p* ≤ 0.001). The picture about deviant probability is less clear-cut. Overall, deviant probability counterbalances the negative effect of deviant position and distance: as probability of encountering a deviant increases (i.e., later in a sequence), RTs also increase (*t* = 7.87, *p* ≤ 0.001). Considering that deviant probability depends on position, these two effects compensate each other (this is visualised in Supplementary Figure 2, where we plot model predictions separately for each term in the model). Note that, although there is no main effect of condition, there is a statistically-significant interaction between condition and deviant probability. As we observe in Figure 2, condition 3 shows the steepest curve of all, which corresponds with the fact that the slowing effect of deviant probability is diminished for condition 3 (*t* = −3.18, *p* = 0.002).

**Table 2:** Summary of the fixed effects in the final mixed model analysis (dependent variable = log-transformed, z-normalised reaction times).

**Term**	**Estimate**	**Lower ***CI*****	**Upper ***CI*****	*****SE*****	*****t*****	*****p*****
(Intercept)	0.77	0.46	1.07	0.16	4.90	<0.001^***^
deviant.position	−0.22	−0.25	−0.19	0.01	−15.74	<0.001^***^
deviant.distance	−0.01	−0.01	0.00	0.00	−3.88	<0.001^***^
deviant.probability	1.78	1.34	2.23	0.23	7.87	<0.001^***^
condition2	−0.02	−0.42	0.39	0.21	−0.08	0.939
condition3	0.09	−0.30	0.48	0.20	0.46	0.648
deviant.probability: condition2	−0.44	−0.91	0.03	0.24	−1.82	0.069
deviant.probability: condition3	−0.73	−1.18	−0.28	0.23	−3.18	0.002^**^

*Significance symbols:*

** *p ≤ 0.01;*

*** *p ≤ 0.001*.

## 4. Discussion

Overall, our experimental results accord with the Strict End Hypothesis posited for verse: (1) later in the verse line, deviations are less frequent, and (2) later in the experimental sequence, deviations are detected faster. Nevertheless, the three conditions under inspection do not pinpoint the defining rhythmic context under which the decreasing reaction time takes place.

The regularity of ISI and ITI in condition 1 entails that the timing of the events is maximally predictable. In condition 2, the sounds within sequences are regularly spaced, but the onset of each sequence is unpredictable. Hence, by comparing conditions 1 and 2, we test whether the crucial factor producing a decrease in reaction time lies in the uncertainty of knowing *when* the first stroke of the sequence will be heard. An unpredictable beginning would produce a sequence-initial disadvantage, which would then disappear as further strokes are played with predictable timing. The results do not show a difference in initial disadvantage between conditions: both conditions show an initial disadvantage, even if the onsets of sequences in condition 1 are completely predictable.

Given the between-groups design, however, an important caveat is in order. The reported analysis is based on z-normalised RTs as the dependent variable (normalised within each experimental setup). The raw RTs (Supplementary Table 1) show that condition 3 is fastest. This could in fact be due to the experimental manipulation, although this would suggest that the least-predictable condition provides a processing advantage. Condition 3 involves variable ISI, making it less predictable than condition 2, where the unpredictability only involves the initial onset of each trial. Having variable onsets for every stimulus may prove a more challenging task, requiring sustained focused attention; if such focused attention is in fact triggered by the exceptional demands of unpredictable ISIs, this attentional adjustment might in fact improve task performance and thus account for the observed RT differences between conditions.

Future research using a within-subjects design will help eliminate the group-condition confound, while keeping a unique setup will deal with the machine-condition confound. The current design, hence, is only suited to capture relative differences affected by the predictor variables (i.e., deviant position, distance, and probability). In this respect, we observe, for instance, that deviant probability tends to increase RTs, but not equally under all conditions. The effect is most pronounced under condition 1, and less so for the other two conditions (see Supplementary Figure 2C). Condition 1 (constant ISI and ITI) could, in principle, have a global advantage by virtue of its regularity, but the present study only addresses relative differences in RTs, not absolute ones.

Unlike similar oddball experiments (Schwartze et al., 2013; Bouwer and Honing, 2015), our stimuli were organised into sequences separated by a longer silent gap. To be sure, the stimuli used in Bouwer and Honing (2015) have a recurring metrical structure which does evoke eight-beat sequences. Nevertheless, and despite the differences in design, we can hypothesise that the crucial difference which produces the decreasing reaction times lies in the sequence-dividing silent gaps. At the very least, we can conclude that the manipulation of ISI or ITI regularity did not eliminate (or diminish) the main effect of deviant position: in all three conditions, reaction to a deviant became robustly faster as it got further away from the preceding ITI pause.

This is in line with early experimental evidence by Stetson (1903), who also used pseudo-verse stimuli. Subjects listened to sequences of eight regular clicks; as in our experiment, sequences were separated by a longer interval. Their task was also to detect irregularities; not deviant sounds, but deviant ISIs, that is, lags or longer-than-expected intervals between ticks. Subjects were much more accurate in detecting these irregularities if they occurred late in the line (between ticks 6 and 7) rather than early in the line (between ticks 2 and 3). Further manipulations of the kind led (Stetson, 1903, p. 421) to conclude that the results provided evidence for “the increased exactness of the rhythmic perception at the close of the verse.” Due to the temporal nature of his manipulations, Stetson (1903) did not test irregularities similar to our conditions 2 and 3, but focused instead on the kind of regularity found in our condition 1 (constant ISI and ITI).

Contrary to the global initial disadvantage recorded in our three conditions, Cowan et al. (1993) did observe a greater initial disadvantage in the unpredictable condition, compared to a more predictable one. The experimental design differed in several aspects, though. Sequences of nine tones were presented, with deviant tones occurring in varying positions within the sequence. The gap between sequences, though, was approximately ten times longer than in our study (between 11 and 15 s). The experiment analysed Event-Related Potentials (instead of RTs), and the analysed outcome was the Mismatch Negativity (MMN) generated in response to the deviants. After three or four standards, an MMN was consistently observed; at the very beginning of a sequence, however, deviants did not generate an MMN response. Crucially, in their more regular (hence predictable) condition, a single standard at the beginning of a sequence was enough to produce an MMN after the second tone; in their unpredictable condition, though, more than one standard was needed to set up the relevant representation which could then be violated and generate an MMN. Regardless of regularity, the three conditions in our study show a sharp drop in RT after the very first position. Similarly, other studies have claimed that, contrary to Cowan et al. (1993), auditory regularities are not needed for an MMN to be generated (Jääskeläinen et al., 2004; Campbell et al., 2007).

The predictability-driven initial disadvantage (and final advantage) relies on the general readiness principle: if one can predict *when* an event will occur, the speed and accuracy with which we respond to the event is enhanced (Woodworth, 1938; Niemi and Näätänen, 1981). Despite the difference between our experimental conditions, the longer gap which precedes sequences in all three cases can be interpreted as a disruption of readiness.

Beyond readiness, finer-grained models of how attention is modulated as a function of predictability become relevant. According to the dynamic attention model (Large and Jones, 1999; Large et al., 2010), when we track an external regular rhythm such as a beat sequence, our attention is modulated at the same rate as the rhythm via entrainment. Empirical work (Jones et al., 2002; Jongsma et al., 2004; Fitzroy and Sanders, 2015) has shown that performance (a proxy for attention) peaks at the points where a beat is predicted, and decreases elsewhere. As the underlying mechanism, it is hypothesised that neural populations synchronise to the external rhythm by firing at the same rate.

The dynamic attention account can explain an increasing advantage later in the line, as the neural entrainment comes into place and attention tracks incoming strokes more precisely. Note that, under this model, the overall level of entrainment (reflected by the oscillator amplitude) is lower under conditions 2 and 3, due to their temporal irregularities. Nonetheless, the model is robust enough to these perturbations of isochrony, and it predicts an acceleration of reaction times in all three conditions. While the decreasing-RT slope would be similar, the irregular conditions are predicted to show higher RTs overall under this model. Our current experiment, however, does not tackle the issue of absolute RTs, but only their relative variation within sequences.

An alternative model which can account for the data is the Bayesian predictive coding model (Vuust and Witek, 2014). In this case, the prediction of events gets continuously updated as new stimuli are processed, regardless of isochrony. New strokes of the same kind reinforce our prediction, deviants become more disruptive as a consequence, and sensitivity to these violations of the standard gets enhanced.

Further simulation studies are needed in order to evaluate more precisely the extent to which these (and other) models fit the data at issue. An adequate model for final strictness in verse may rely on regularities of the input, though preferably not too strictly on a stringent temporal regularity. This has the potential of being applicable to a broader range of verse types, not restricted to prototypical metrical songs (where a regular beat can be felt), but including, for instance, non-isochronous poetry recitation.

Fluctuations of attention across verse lines offer a possible explanation of final strictness defined as a decrease in the probability of deviations. Nevertheless, there exist other phenomena related to final strictness, such as rhyme, or the categorical control of word-length at the end of the line (Fabb, 2002, p. 174). An increasingly efficient use of attention is not well-suited for these other kinds of final strictness, where the very end of the line is targeted. We should conclude, instead, that verse final strictness is driven by a variety of factors, including attention, the highlighting of constituent boundaries, or facilitating retrieval from memory (Rubin and Wallace, 1989; Rubin, 1995).

Further work is required in order to bridge two critical gaps. First, the low-level oddball task used here should be followed up with more ecological stimuli using e.g., verbal material (i.e., syllables), and rhythmic sequences. Many poetic metres, for instance, rely on the alternation of strong and weak positions; hence, deviations from the norm in that kind of context are more complex than in the present paradigm, where violations deviate from a single standard tone. Second, the gap between perception and production needs to be addressed, since the final strictness evidence which motivated the study relies on how poets and singers *produce* their lines of verse, not on how they *perceive* them. Unavoidably, the extent to which the attention mechanisms described here are applicable in a comparable way during the generation of lines (or other non-linguistic sound sequences) needs to be determined by production experiments.

## 5. Conclusion

Versification systems are cultural phenomena shaped by a complex interaction of factors. Typological tendencies such as final strictness can shed light on some of the underlying principles which both make possible *and* constrain the production of songs and poems. When the subjects in our experiment were asked to track the sequences of drum strokes and react to the deviant ones, their performance consistently dropped after the sequence-dividing gap. We propose that a similar drop of attention can play a role in the reduced faithfulness to templates found in songs and poems. Nonetheless, it should be kept in mind that, besides cognitive or anatomical constraints, verse is also shaped by aesthetic ideas which purposefully satisfy and violate our expectations.

## Data Availability Statement

The dataset presented in this study can be found in the following online repository: https://github.com/vdca/seh2.

## Ethics Statement

Ethical review and approval was not required for the study on human participants in accordance with the local legislation and institutional requirements. The patients/participants provided their written informed consent to participate in this study.

## Author Contributions

VDCA: conceived and designed the experiment, gathered and analyzed the data, and wrote the paper.

## Conflict of Interest

The author declares that the research was conducted in the absence of any commercial or financial relationships that could be construed as a potential conflict of interest.

## Publisher's Note

All claims expressed in this article are solely those of the authors and do not necessarily represent those of their affiliated organizations, or those of the publisher, the editors and the reviewers. Any product that may be evaluated in this article, or claim that may be made by its manufacturer, is not guaranteed or endorsed by the publisher.

## Footnotes

^1^Specifically, the standard sound is the *ta* stroke with identifier 224350, and the deviant tone is the *num* stroke with identifier 224279; both have a fundamental frequency of 116 Hz.

^2^Normalising RTs by participant leads to the same pattern of results. Hence, the possibility of the results being driven by setup differences remains unlikely.

^3^We have tested whether accuracy is affected by experimental condition or by any of the three deviant-related variables (position, probability, distance) through a mixed logistic regression. We have applied the same stepwise model-selection procedure as described in section 2.3, with response accuracy as the dependent variable, and using the LMERConvenienceFunctions package (Tremblay and Ransijn, 2020) in R. The final model only includes deviant probability as a fixed effect, although without reaching statistical significance (*z* = −1.68, *p* = 0.092). Hence, we exclude that the observed effect of deviant position on RTs merely reflects a tradeoff between speed and accuracy.
