# Supplementary material for: Deviants Are Detected Faster at the End of Verse-Like Sound Sequences
Source: Front Psychol. 2021 Aug 31;12:614872. doi: 10.3389/fpsyg.2021.614872 (PMC8438167; doi:10.3389/fpsyg.2021.614872)
Supplement: Supplementary file 1 [file Data_Sheet_1.PDF]

Supplementary Information

condition	RT (ms) M	SD	subjects	observations	accuracy (%) M	SD
1: constant ISI & ITI	580	121	13	586	97.6	1.66
2: variable ITI	569	121	15	677	96.7	2.97
3: variable ISI	391	85	18	828	97.9	2.33

Table S1. Descriptive statistics summarising the 2,091 RT observations used in the analyses.

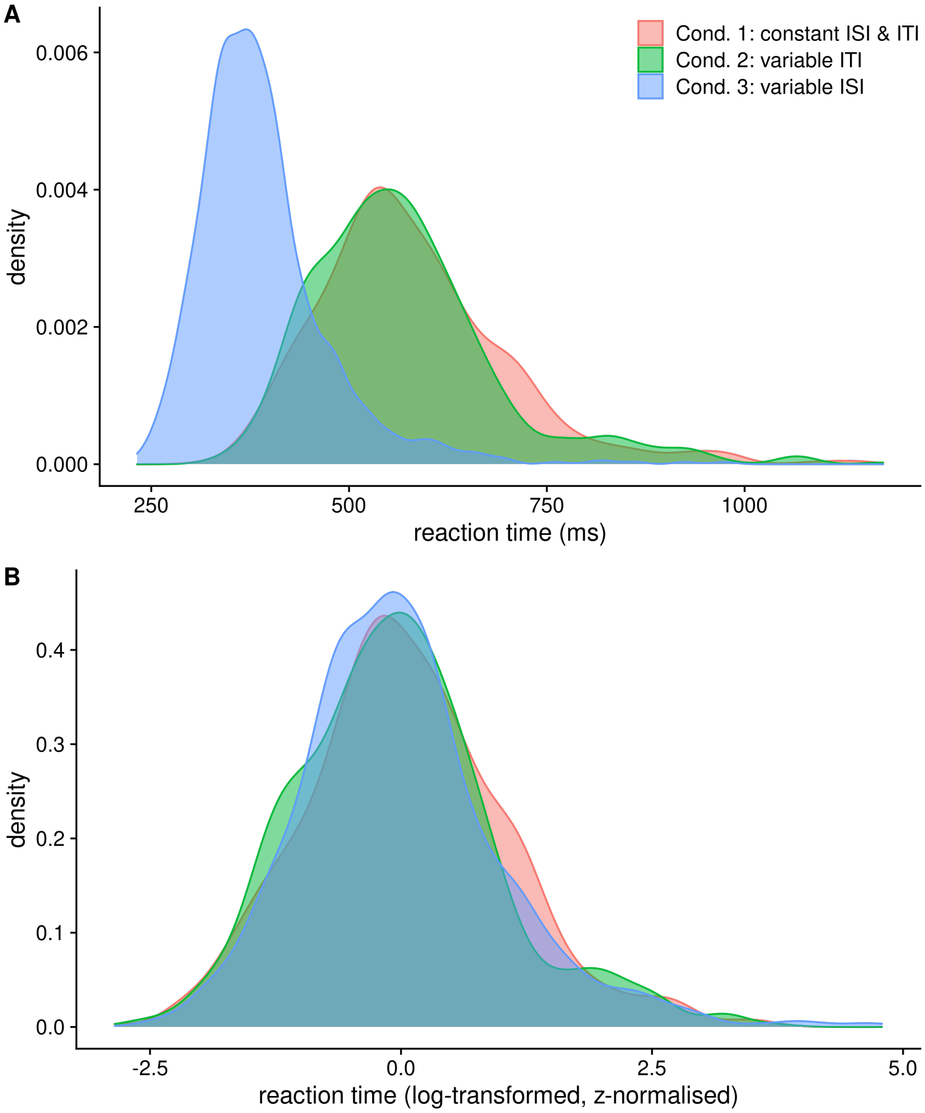

Figure S1. Distribution of reaction times (RTs). **A**: non-transformed RTs in milliseconds. **B**: log-transformed RTs, z-normalised by experimental setup (hardware and software affects RT lags).

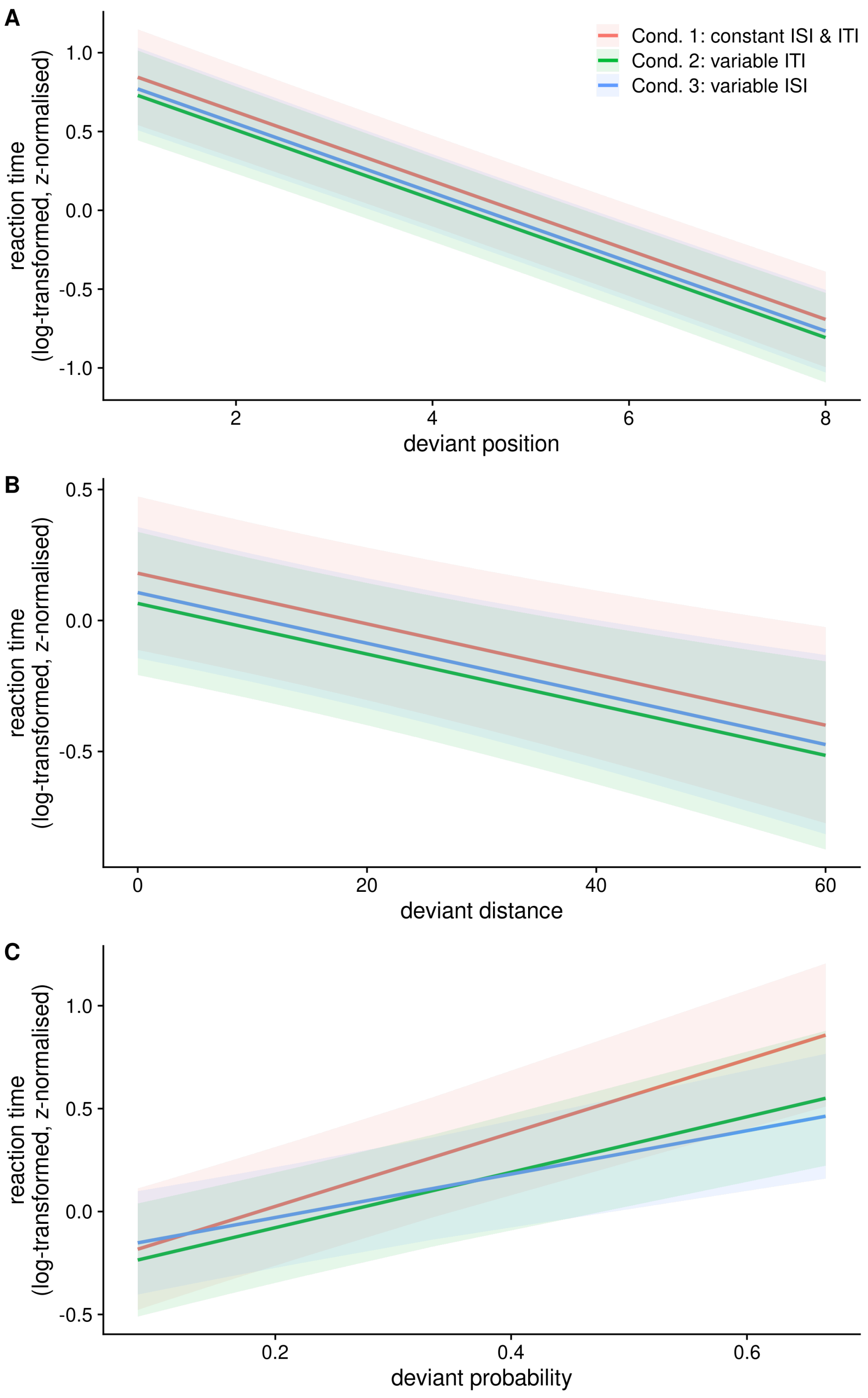

Figure S2. Model predictions for each of the fixed terms included in the final model (see Section 3 and Table 2 for details). Note that all three main effects are significant, but the interaction with experimental condition is significant only for deviant probability (**C**).
